# “Laying bricks to build integrated career guidance plans”: Best practices in vocational education and training in Andalusia, Spain

**DOI:** 10.3389/fpsyg.2022.1001836

**Published:** 2022-09-15

**Authors:** Soledad Romero-Rodríguez, Celia Moreno-Morilla, Tania Mateos-Blanco

**Affiliations:** ^1^Department of Research and Assessment Methods in Education, School of Education, Universidad de Sevilla, Seville, Spain; ^2^Department of Theory and History of Education, and Social Pedagogy, Universidad de Sevilla, Seville, Spain

**Keywords:** career development, career guidance, Vocational Education and Training (VET), education system, comprehensive career guidance

## Abstract

Vocational Education and Training (VET) policies are paying increasing attention towards the need to develop quality career guidance processes. Career guidance plays an essential role in this challenge by enabling people to develop the career management skills that allow them to become aware of their full potential and to construct life projects that facilitate the emotional management of uncertainty and complexity, participating as responsible agents in their environment. After conducting a literature review on each of these key elements, we set out to identify best practices in VET as “building blocks” for the construction of an integrated career guidance plan in VET. This study adopts an approach based on qualitative methodology, specifically a documentary type of research has been carried out. The documents analysed correspond to 88 best practices identified in Andalusia (Spain). The analysis strategy used was a content analysis. For this purpose, a system of categories has been designed, from a deductive perspective, which has contributed to a systematic analysis of them. One of the first conclusions of the study is the uneven development o best practices in the different VET professional pathways. The practices carried out in the centres begin to incorporate different elements and thus facilitate the creation of a suitable context for the development of comprehensive career guidance plans in VET. The article closes by offering implications for the development of guidance in the education system (at VET level) and discusses lines of future research.

## Introduction

VET policies are paying increasing attention to the need to develop quality career guidance processes. This is highlighted in the *Council Recommendation on Vocational Education and Training* (*VET*) *for Sustainable Competitiveness, Social Fairness and Resilience* (2020/C 417/01) and in the *Osnabrück Declaration on VET as an enabler of recovery and just transitions to digital and green economies* (30 November 2020). Education systems must provide VET that equips people with the necessary competences to build their careers and manage transitions, fostering inclusion and equal opportunities and contributing to resilience, social justice and sustainable development. Career guidance plays an essential role in this challenge by enabling people to develop career management skills that allow them to become aware of their full potential and to construct life projects that facilitate the emotional management of uncertainty and complexity, participating as responsible agents in their environment.

The different reports and recommendations that have been developed by European bodies have justified the need to develop comprehensive career guidance systems by alluding to the benefits they could bring as a requirement to offer inclusive, quality guidance linked to lifelong learning (Comisión Europea, 2004, 2008, 2018; CEDEFOP, 2006, 2008, 2010). The comprehensive nature of guidance intervention (Gysbers and Henderson, 2014) involves bringing together both the principles of prevention, development and social intervention and the different aspects of career management guidance (personal, academic, vocational). In order to develop a guidance intervention of these characteristics, a series of conditions must be met, such as: integrating the training in competences incorporated in the curriculum with the specialised work of the guidance counsellors; taking advantage of all the resources available inside and outside the educational centre; promoting the participation of all the agents and acknowledging the prominent role of the ultimate addressees, i.e., the students. This systemic structure of guidance intervention, its internal coherence and the involvement of all actors and stakeholders are the main contributions of a comprehensive guidance model (Rodríguez-Diéguez, 2008).

In a recent study (Romero-Rodríguez et al., 2020b; Romero Rodríguez et al., 2022) we used a collaborative research methodology to encourage different stakeholders and users to propose challenges and actions to develop comprehensive career guidance systems in VET. The outcome was the proposal of a set of challenges, for which 28 objectives and 57 actions were formulated to address them. Table 1 lists the challenges that referred to the practices of the VET centres.

**Table 1:** Challenges for the development of comprehensive career guidance in VET.

Planning of career guidance integrated into the curriculum throughout schooling. Development of the integrated vocational information and guidance system. Attention to the success and return of Vocational Education and Training students. Improving knowledge and awareness of Vocational Education and Training (VET). Development of experiential methodologies for vocational training and guidance. Fostering collaboration of companies.

Each of these challenges refers to a key element for the elaboration of integrated guidance plans: career competencies, collaboration, attention to drop-outs, knowledge of VET, methodologies, experiences and involvement of companies. Other challenges considered to be the responsibility of the administration were: (a) the adequacy of VET planning and academic management; (b) the development of VET quality assurance systems and (c) the adequacy of training and selection of guidance officers (Romero-Rodríguez et al., 2020a). In order to provide research support for the implementation of the proposed actions, a new research project was developed with the aim of analysing good practices in VET that included at least one or some of the key elements among their actions.

This article aims: (1) to identify best practices that can help to build (as if they were “bricks”) comprehensive guidance plans that contribute to the development of career management competences by VET students and to prevent VET early-leaving. Within the framework of our study, the selection of best practices has focused on those which are being developed in Andalusian VET and which address some of the challenges for the development of integrated guidance plans. To this end, the study also aims: (2) to analyse the characteristics of best practices (vocational families involved, target VET levels, objectives and types of actions and contribution to guidance) as well as (3) the relationships between the various key elements.

## Theoretical framework

### Development of career skills in VET

The evolution of VET is moving towards the development of sustainable careers and futures, from an inclusive and socially fair perspective (Observatorio de la Formación Profesional en España, 2021). This approach requires a close relationship between stakeholders and teaching-learning environments, and a commitment by all in the design and planning of curricula and guidance systems (Romero-Rodríguez et al., 2020a, 2020b). In this line, the VET of highly qualified professionals equipped with knowledge and skills for their insertion in productive and social life requires the development of comprehensive training that places the development of the so-called “soft-skills” at its cross-cutting and central point (Ashford, 2019; McDonough, 2020). These skills are a central element in the overall skills landscape, as well as in workplace learning and VET (Aarkrog and Wahlgren, 2017). The literature deeply supports and deploys the need for people to actively build their careers to be employable (Eby et al., 2003; Forrier et al., 2015) and to do so, they need to acquire soft-skills. Moreover, these competencies become strong predictors of success in academic and career transitions (Blokker et al., 2019).

In short, the soft-skills approach contributes to the development of the so-called *career competencies* defined by Akkermans et al. (2013, p. 249) as knowledge, skills, and abilities central to career development, which can be influenced by the individual, and consist of three domains: reflective, communicative and behavioural career competencies. The first ones are related to self-awareness, identification of motivations, strengths, weaknesses, threats and opportunities with regard to one’s career. The second refers to one’s ability to communicate and to communicate with actors and in diverse contexts to improve the chances of success. The third ones include the ability to explore the work environment efficiently in search of their own employability, as well as the planning of career goals (Savickas, 2005; Forrier et al., 2015; Blokker et al., 2019). The development of comprehensive training and guidance empowers individuals to engage with their training, to progress in qualification and lifelong learning and to cope with different personal and work transitions (ELPGN, 2015).

### Collaboration of guidance counsellors

Career constructing processes, far from being an exclusively individual responsibility, imply social responsibility of private organisations and public administrations, thus representing an opportunity for community development (Sultana, 2018). Different bodies and entities with responsibilities in public training and employment policies have tried to showcase the importance of this collaboration. The joint work carried out by the International Labour Organisation (ILO) and European Training Foundation (ILO & ETF, 2021) and by EU, ETF, CEDEFOP, OECD, ILO, and UNESCO (2021) emphasises the need for collaboration and cooperation among stakeholders from system review until the development of national action plans. Among the groups that should co-operate with each other are “representatives from education, employment, youth, health, economy and stakeholders from wider civil society” (ILO and ETF, 2021, p. 38), including career services, business community, representatives of government, employers, trade unions, youth organisations, NGOs and community organisations (ETF, 2020).

However, despite the importance given to the development of integrated systems (Manzanares Moya, 2013), research (CEDEFOP, 2010) highlights the fragmentation of services (Watts, 2014), which is greater in countries with decentralised administrations, as is the case in Spain. Some studies carried out in Spain (Blázquez et al., 2019) conclude that for there to be collaboration between administrations, companies and entities, it is necessary to make training content more flexible so that it can respond to the needs of the labour market and prepare young people for a satisfactory professional career.

### Prevention of early school leaving in VET

One of the problems the education system faces, in general, and VET in particular, is the high percentage of students who leave school prematurely. As reported in the, Eurydice (2014), CEDEFOP (2014), and CEDEFOP (2017) reports, several initiatives have been developed to fight against early school leaving (ESL) in education from different approaches: prevention, intervention and compensation. Currently, the European Commission (2020) is especially committed to activating prevention by means of early warning systems for ESL and through strategies centred on professional guidance, the development of more flexible learning pathways, the use of active methodologies and generating learning processes in the workplace.

Best practices have been developed that focus on early detection of the problem in lower-level VET (Bates et al., 2015) with a key aspect of this process being the availability of tools that enable professionals to identify cases at an early stage (Pinto Teixeira et al., 2014; Blaauw et al., 2019). CEDEFOP (2017) presented a toolkit called *VET Toolkit For Tackling Early Leaving*^1^ which provides a European-wide resource bank to promote practical initiatives to combat ESL in Vocational Education and Training. As a resource proposed from the intervention approach it is recommended to support young people to acquire career management skills through guidance so that the learning and working life choices they make are sufficiently motivated and successful.

One of the preventive approaches that has gained prominence in recent years is the focus on vocational training experiences linked to the concept of student engagement. From this type of practice, the emotional, behavioural and cognitive dimensions of the students are worked on through more individualised guidance processes. This ensures that students study professions with which they have some emotional and/or instrumental connection (Cerdà-Navarro et al., 2019; Cerda-Navarro et al., 2020; Olmos-Rueda et al., 2020).

### Awareness of Vocational Education and Training

Despite the growing appreciation of VET linked to employability, the different countries in Europe continue to focus on increasing the attractiveness of this training pathway as a choice for post-compulsory studies. Several studies (Pimpa and Suwannapirom, 2008; CEDEFOP, 2014; Cao and Thi Tran, 2015; Chankseliani et al., 2016; Ajithkumar and Pilz, 2019) show that, despite the improved image of VET, families and school staff themselves still do not see it as a priority option, except for those who have taken it.

With the aim of improving the social perception of the attractiveness of VET, a European-wide initiative such as WorldSkills^2^ has emerged, in which young VET students or recent graduates take part. The aim of this global competition is to raise the level of excellence and recognition of VET as a professional pathway. Although it is a valuable event in the field of VET, the actual impact on the improvement of its image is not as expected. As Chankseliani et al. (2016) warn, policy efforts are needed to disseminate excellence to the entire VET sector, and to publicise these events more widely as an opportunity to make the training of young people visible and more widely recognised. The role of the media as a valuable instrument for promoting VET and as a means of stimulating students, teachers and companies should also be considered (Di Pietro, 2018).

However, when the aim is to work in a closer context with students and their environment, the proposals are made within VET centres and are mainly limited to generating learning environments of an experiential nature (link between the world of training and the labour market) and socialisation for work (Aiello, 2020; Rintala and Nokelainen, 2020).

### Active and experiential methodologies in VET

Scientific literature as well as official documents in the field of education underline the need to “improve education and the updating of assessment methods as well as innovative forms of teaching and learning” (Council Recommendation on key competences for learning, 2018 p. 189/2). Some of the proposed methodological strategies emphasise the development of interdisciplinary and collaborative learning, challenge solving and problem-based learning, student decision-making and a stronger connection to the professional sphere (Numonjonov, 2020).

Experiential learning methodologies favour the construction of the students’ life and professional project (career construction; Sultana, 2004; CEDEFOP, 2008). Some tools used in different European countries, as reported in different reports of CEDEFOP (2008), to facilitate the self-reflective process are portfolios (which can be electronic or virtual). In order not only to broaden career and employment options, but also to foster the development of transferable skills in a more transparent manner, experiences with the labour market may include work contexts other than the profession for which they are preparing.

The research results also show how gamification is becoming a key element in VET. The inclusion of gamification, accompanied by badges, levels, leader boards, rewards, etc. generates greater motivation and commitment among students to actively participate (Garcia-Iruela et al., 2021), and there is evidence of a great deal of best practice in this regard.

### Involvement of companies

We understand “employer engagement in education” as described by Musset & Kureková (2018, p. 68):

The engagement of people from across the economic community who exchange labour for financial rewards with children and young people as mediated by their schools and colleges. Consequently, the idea of employer engagement is as just as important to professional associations and trade unions as it is to employers themselves.

When we talk about collaboration with companies, we are referring to entrepreneurs’ associations, trade unions, non-governmental organisations, scientific and teaching communities and heads of VET institutions (Terentyeva et al., 2018). The recent report by EU, ETF, CEDEFOP, OECD, ILO, and UNESCO (2021) noted the importance of this involvement as an essential element of career guidance processes. In contrast to the idea that corporate involvement can be a tool for the reproduction of social inequalities, Percy and Kashefpakdel (2018, 2021) argue that, if used in a deliberate way, it can help people re-imagine other roles and overcome social barriers.

A recent report of OECD (2021, p. 1) has indicated the objectives to which this involvement should respond in relation to career guidance, including to enhance their understanding of jobs and careers, to provide them with the knowledge and skills demanded by the contemporary labour market and for successful school-to-work transitions, and underpinning pupil attainment. This will bring a range of benefits for students, such as offering selection criteria for jobs, attitudes to work and relationships, career choices and plans and specific work-necessary skills (Watts, 2015). For their part, companies also benefit, namely by the identification of future skills training needs, the opportunity to promote careers in their professional sector, the opportunity to meet potential candidates before they are recruited or to improve the employability of young people who will be more effective workers in the future (Hooley et al., 2015).

Some authors have looked at the characteristics that corporate involvement should have (Mann et al., 2018a,b): authentic, frequent and often mandatory, valued, varied, contextualised, personalised and begun at a young age. Others (Gessler, 2017) propose a conceptual framework of collaboration in which a formal structure is defined that should develop systemically through day-to-day interactions (Percy and Kashefpakdel, 2021).

## Materials and methods

This research follows an approach based on qualitative methodology, specifically a documentary type of research has been carried out. The documents analysed correspond to the good practices identified (Objective 1) by the Directorate General for VET of the Andalusian Regional Government in a specific call that highlights innovation, the application of active methodologies and internationalisation in VET. In this paper, 88 of the 104 best practices selected in the 2019–2020 call are analysed. The selection of these practices is based exclusively on the fact that they contribute to the development of guidance from a perspective of improvement and innovation. Practices that included some of the key elements described in the previous section were selected.

The analysis strategy implemented (Objective 2) was to carry out a content analysis. To this end, a system of categories has been designed, from a deductive perspective, which has contributed to a systematic analysis of the categories. The identification of themes was carried out independently by the three researchers in order to triangulate a categories system for coding the content itself. Table 2 shows the categories system used.

**Table 2:** Category system.

Category	Definition
Career skills development	Actions aiming to develop career competences
Collaboration of guidance counsellors	Actions in which different internal and external guidance counsellors collaborate (educational and labour administration, social agents, families, students, etc.)
Attention to drop-outs	Actions to prevent, or act upon, situations of early drop-out from the educational sphere
Knowledge and awareness of VET	Activities aiming to improve the knowledge and social image of VET
Experiential methodologies	Educational actions using active methodologies, centred on the students and their personal experience
Involvement of companies	Training and/or guidance activities developed with the participation of businesses (public, private, third sector).

A double-entry table was constructed in which the 88 best practices were included and the key elements they contained were identified. Although the methodological approach has been eminently qualitative, a multiple linear regression model has also been run to analyse the effect of the key elements’ collaboration, knowledge and awareness of VET, experiential methodologies and company involvement on what could be considered three desirable outcomes: the development of career management competences, attention to drop-outs and knowledge of VET (Objective 3). Cross tables and a Chi-square analysis were also carried out to study the association between the different elements analysed.

## Results

### Identification of best practices (Objective 1)

**Figure 1:**
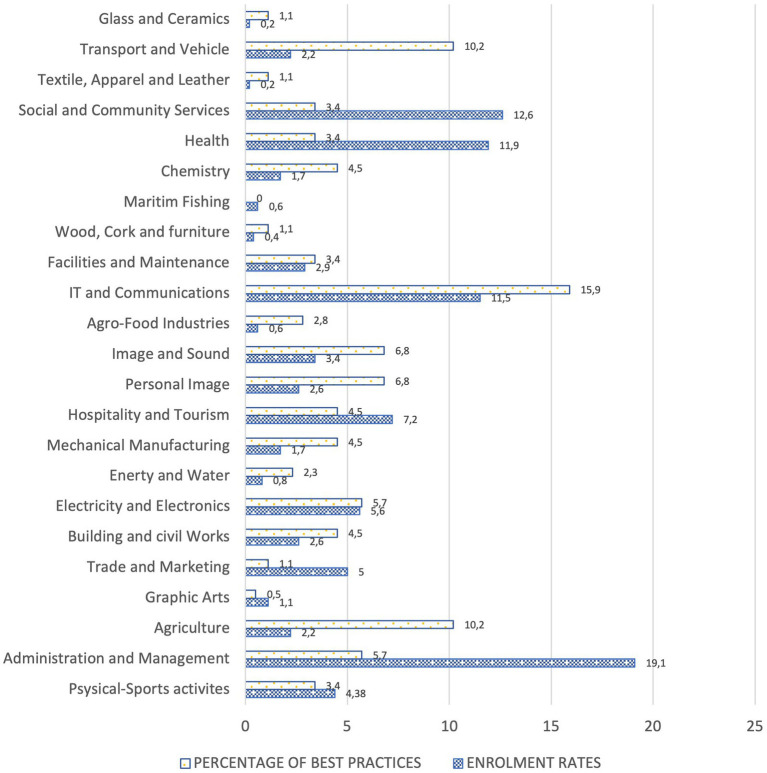
Enrolment rates and percentage of best practices by professional groups.

One of the first findings of the study is the uneven development of best practices in the different VET professional families. As can be seen in Figure 1, firstly, there are some professional families that stand out for the high percentage of best practices identified. Particularly noteworthy is the case of Information Technology and Communications. On the other hand, it can be observed that there are professional families in which, despite having a lower enrolment rate, they develop a higher percentage of best practices (e.g., Agriculture; Transport and Vehicle Maintenance; Image and Sound; Personal Image; Glass and Ceramics). Conversely, there are professional families in which, despite offering a wide variety of training cycles and a large number of places, only a small number of best practices have been identified. In this sense, the families of Health, Social and Community Services, Administration and Management or Hotel and Tourism Management or Hospitality and Tourism stand out.

In the module of Training and Career Guidance (*Formación y Orientación Laboral*-FOL in Spanish), a percentage of best practices (13.6%) is observed which, although higher than that of most of the professional families (except Computer Science and Communications), it is considered low if we take into account the guidance function of this module and its cross-cutting nature to all vocational families.

**Figure 2:**
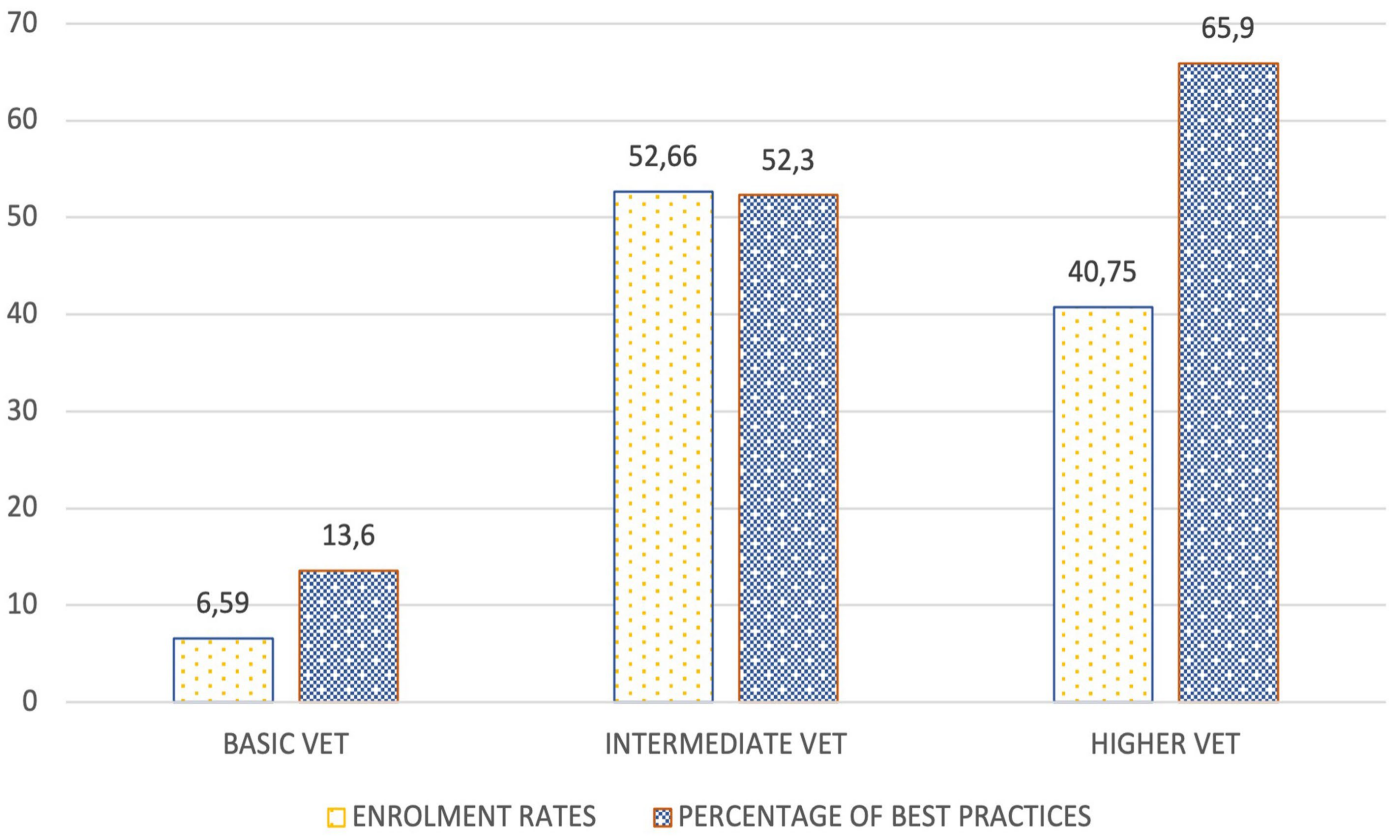
Enrolment rates vs. Percentage of best practices.

With regard to the level of VET at which they are developed, as can be seen in Figure 2, those aimed at the Higher Level Training Cycle (*Ciclo Formativo de Grado Superior*-CFGS in Spanish) prevail (65.9%), being less present at the Basic Vocational Training (*Formación Profesional Básica*-FPB) level (13.6%). However, this presence should be seen as consistent with the volume of enrolment at both levels. In any case, and in comparison with the Intermediate (*Ciclo Formativo de Grado Medio*-CFGM) Level (52.3%), it can be observed that there is a higher proportional incidence of good practices in the Higher Level.

**Figure 3:**
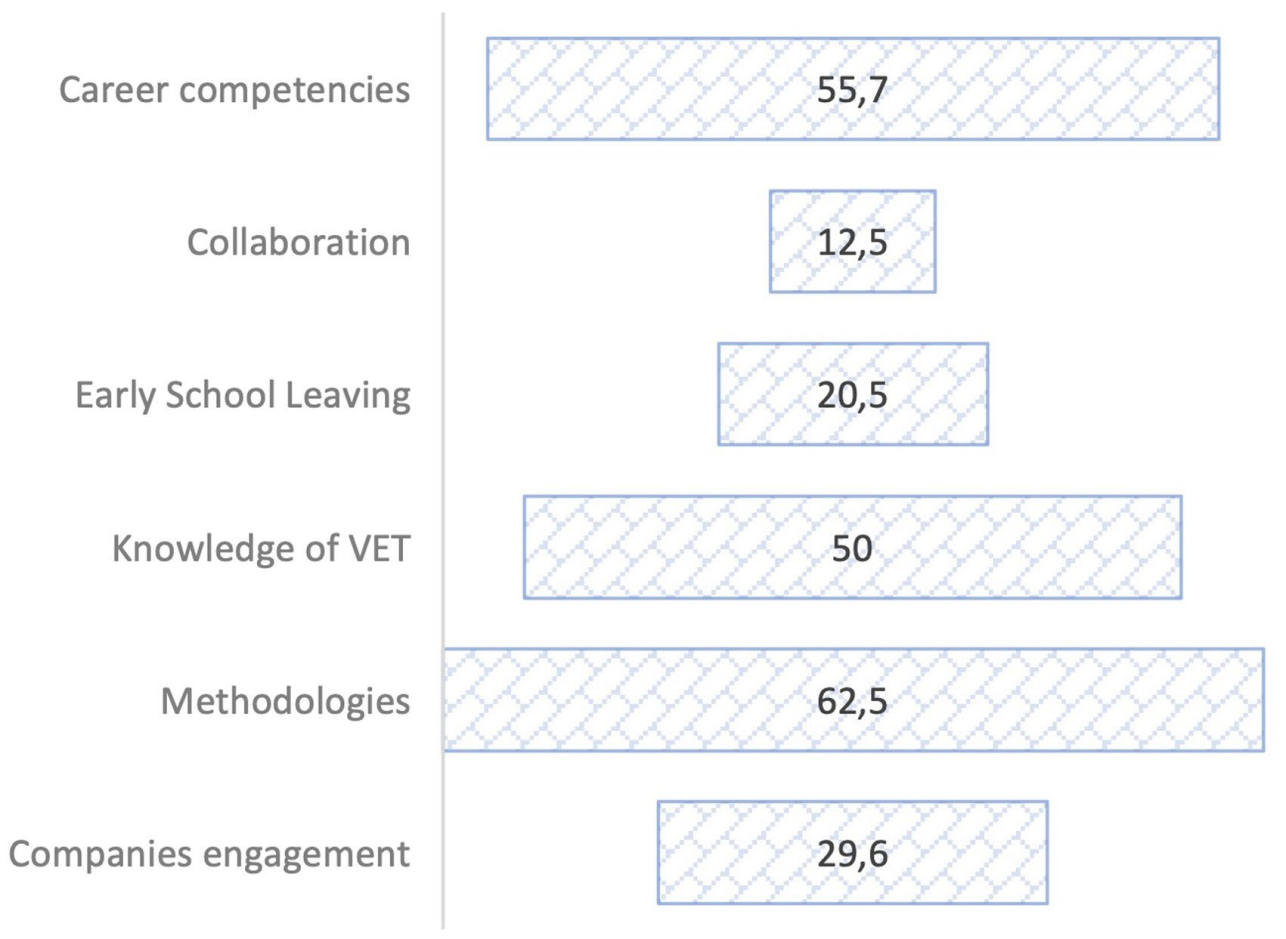
Best practices x Key element.

Finally, Figure 3 shows that apprenticeships in the centres do not comprehensively cover all the key elements. However, they are beginning to incorporate some of them and thus facilitate the creation of a suitable context in which integrated VET guidance plans can be developed. There is also unequal attention in terms of the different key elements (see Figure 3), with the use of experiential methodologies being those with the highest presence (62.5%). The element that received the least attention was the actions to foster collaboration between different guidance counsellors (12.5%).

### Analysis of the content of good practices: Examples of good practices (Objective 2)

#### Innovating towards comprehensive training and career skills development

Actions contributing to the development of comprehensive training are identified in 55.7% of the selected best practices. These apprenticeships are mainly developed by the professional families of IT and communications (8), Image and sound (6) and those promoted by FOL (6). Higher level education is the one that encourages this type of apprenticeship the most (37.5%), followed by middle-level education (33%) and, lastly, Basic Vocational Training (9.1%).

From the point of view of the development of comprehensive training and guidance in VET, the analysis of the selected practices reveals some common goals (see Table 3):

**Table 3:** Creating a show in the classroom: Talent show project.

Professional family: Image and sound
Development location: IES Ángel de Saavedra Secondary School (Córdoba, Andalusia)
This experience aims to create a real project of inter-cycle shows and events in VET with the main objective of enabling students to plan and manage the production of shows and events. This experience also aims to familiarise students with the functions and responsibilities of the different roles in work, learning to interpret the technical and artistic documentation necessary in the creation of stage spaces and to coordinate the staging. In addition, committed to the development of sustainable careers, this experience aims to encourage cooperative work and raising resources to donate to an NGO in Córdoba.
**On-site**
Professional family: Installation and maintenance
Development location: IES Tres Molinos (Huelva, Andalusia)
This innovation was developed in response to the needs and difficulties presented by the students studying VET. It was considered that the use of this method could reduce the difficulties of understanding and internalisation of specific content. The aim of this educational practice is to ensure that students acquire the necessary knowledge for the didactic units that are taught using an organoleptic methodology. As the teachers develop each of the teaching units, it is necessary to carry out a tour outside the school so that the students can appreciate and process *in situ*, through their senses, the contents that have been taught during the teaching unit. During the tour, in a relaxed and enjoyable way, the teachers provide explanations, a battery of questions and answers with debates, thus achieving the involvement and participation of the entire group.
**Youth entrepreneurship with “Bunker InnSpain”**
Professional family: Hospitality and tourism
Development location: IES Tolosa (La Línea de la Concepción, Cádiz)
The proposal consists of giving a new use to these structures, adapting them, among other uses, to unique tourist accommodation, thus responding to the new needs of today’s tourists. The project arises from the need to encourage entrepreneurship among young people and to retain talent, valuing the historical and cultural resources of the immediate environment, analysing and detecting new business opportunities and new emerging market niches in the tourism sector and thus collaborating in the economic revival of the Campo de Gibraltar region. The objectives it pursues are: to encourage creativity, innovation and responsibility, to detect new business opportunities in its environment and to involve the sector companies and the administrations.

- They contribute to the development of a series of competences for the effective management of young people’s life and professional projects. In this sense, some of the competences identified are related to the management of diversity and the complexity of relationships, the recognition of one’s own talent, autonomy at work, optimisation of resources, identification of personal, social and business opportunities and commitment to the environment, among others.
- They aim to increase students’ motivation and involvement in their work, share teaching resources and connect theory with the reality of the labour market, facilitate self-learning and self-assessment of the diversity of talents, as well as the development of communicative, cooperative and creative skills. In the following lines we share some good practice actions related to the development of comprehensive training and guidance in VET.

#### Moving towards collaboration between guidance counsellors

Actions that consider collaboration between the different guidance counsellors are present in only 12% of the selected good practices, showing the difficulties that are present when fostering comprehensive guidance support. They only appear in a disjointed manner in 6 of the 22 professional families analysed (Technical-Sports Activities, Agriculture, Graphic Arts, Building and Civil Works, IT and Communications and Chemistry). It is among those organised by FOL that we found the greatest number of experiences (4). As far as the levels of vocational training are concerned, no good practice is observed in VET, while 80% target either Intermediate Level (CFGM) and Higher Level (CFGS) indistinctly and 10% focus on.

In addition to being present in a small number of actions, the collaboration that is established is often partial and far from what is needed to create integrated guidance systems. However, the efforts made by the centres to establish different types of synergies are to be highly appreciated. The analysis of the collaboration channels established in the selected best practices leads us to observe some characteristics of these synergies:

- Collaborations are established with educational centres of origin of future VET students. The main objectives of the actions carried out within the framework of this partnership are to raise awareness of VET and to accompany the transition.
- Collaboration with business sectors in the area where the centre is located, with the aim of designing Workplace Training (*Formación en Centros de Trabajo*-FCT in Spanish) modules, designing dual training projects or developing talent recruitment programmes.
- Actions are developed to enhance the employability of graduates through the organisation of entrepreneurship and co-working projects in which local entities or CADEs participate.
- Development of Erasmus+ Projects in collaboration with educational centres in other countries. With them, joint analyses of the suitability of the curriculum are carried out and shared teaching units are designed, thus fostering the internationalisation of Vocational Education and Training.
- Use of active methodologies, such as service learning or problem-based learning designed jointly with local entities, companies, neighbourhood associations and third sector involved in the object of these projects (problem or service).
- Efforts towards integration between the education and employment sectors are most evident in integrated educational centres, as in the case of the action “Integrated centre, unique centre.”

The following are some of the actions that illustrate these characteristics of the collaboration (Table 4).

**Table 4:** Integrated centre, unique centre.

Professional family: Chemistry
Development location: C.I.F.P. Profesor José Luis Graiño (Huelva)
VET centre that carries out lifelong guidance processes. The VET classrooms bring together professionals in search of qualification and re-qualification, articulating a space of cooperation between the educational administration, the employment administration and the sectoral productive environment.
**EQVET-Erasmus + Project**
Professional family: Building and civil works
Development location: IES Politécnico Jesús Marín (Málaga)
Erasmus+ Project in which six European countries participate with the aim of improving the mobility and certification of professional competences of students in the professional family. PBL-based teaching units are designed jointly. The design involves educational centres in the six countries in collaboration with institutions and companies operating in the sector.
**Education is geared towards sport**
Professional family: Physical-sports activities
Development location: IES López Neyra (Córdoba)
Organisation of a recreational event involving more than 20 educational centres with students from Primary, Secondary and Baccalaureate levels. The event is based on sporting and tourism-related activities in Córdoba. Different institutions, entities and educational centres collaborate.

#### Motivating the active participation of students in their training project as a measure to prevent early school leaving

Actions aimed primarily at preventing ESL account for 27% of the actions analysed. Most of them showcase the commitment of VET centres in addressing this problem through initiatives to prevent or combat ESL. This type of action has been developed in professional families such as Agriculture, Building and Civil Works, Mechanical Manufacturing, Catering and Tourism, with a particularly significant number in Computer and Communications, Transport and Maintenance and those proposed by the FOL department. The students targeted by these strategies are mostly Higher and/or Intermediate level students (66.6%) and only 33.3% are enrolled in Basic vocational training.

The analysis of the selected actions shows a common line of action to prevent students from abandoning their studies:

- Specific work is carried out with students to develop interpersonal competences and skills (resilience; integration; motivation; self-confidence; autonomous or self-directed learning).
- Strategies/methodologies that generate motivational learning environments (transition programmes; development of experiential methodologies—based on challenges, projects and gamification) and social engagement (service-learning) are promoted.
- Significant efforts are made to adapt training to the reality of the labour and business demands.
- There is an explicit commitment to improve the guidance of the professional pathways: information on professional opportunities and training pathways, on the structure, organisation and competences developed in the Cycle or focused on increasing the expectations of employability.

Below (Table 5), some actions have been selected as activators for the prevention of students dropping out of VET.

**Table 5:** “Aldea a través del tránsito” programme.

Professional family: Agricultural
Development location: IES San Juan De Dios. Medina Sidonia (Cádiz)
Programme in which students from Basic Vocational Training work together with classmates from other educational levels of the same centre. Its objective is to generate a motivating learning environment through outdoor activities where key competences, autonomous learning, integration, as well as values and behavioural habits based on democratic principles are worked on. Among the results achieved, it is worth highlighting that student in Basic Vocational Training are more motivated and integrated into the education system.
**Tools for empowerment in the basic Vocational Training environment**
Professional family: Administration and management
Development location: IES Luis Bueno Crespo. Armilla (Granada)
Project that aims to promote educational experiences to strengthen resilience, optimism, responsibility, collaborative culture and professional autonomy. The results of the experience include the achievement of greater professional and personal autonomy of the students, retention of students from basic vocational training to continue studying intermediate vocational training, as well as an increase in cooperation, collaboration and responsibility that promotes the transfer of knowledge among the students themselves.
**Bartop y Drone**
Professional family: IT and communications
Development location: IES Fidiana. Córdoba
An interdisciplinary initiative aimed at motivating Basic Vocational Training students and preventing them from dropping out by designing a project to assemble an arcade machine with a Raspberry Pi 3. The objective results were a greater involvement of students in their learning process and increased motivation.
**Academic and vocational guidance days (J.O.A.P.)**
Professional family: IT and Communications
Development location: IES Jacarandá. Brenes (Seville)
Holding a conference in which vocational training students themselves take on a direct role in academic guidance at other educational levels, as well as providing information on professional opportunities after completing their studies. With the participation of all the schools in the area, various activities took place: stands, talks, street markets and computer workshops. Among the results of the conference are the motivation of students, the promotion of vocational training and the reduction of school drop-out rates.

#### Creating awareness and acknowledging VET and the talent of its students beyond educational centres

Actions aimed at raising awareness of VET are shown in 50% of the selected practices. The professional families from which they are developed are varied (18 families in total), with Agriculture (6), Image and sound (4) or IT and communications (5) and those actions promoted by the FOL department (8) standing out, especially in terms of number of good practices. In relation to the educational levels of VET, 36.6% are aimed indistinctly at Intermediate and Higher level, 31.8% at Higher level, 13.6% at Intermediate level and only 9% at Basic vocational training.

The analysis of these actions reveals the following strategic lines of action:

- Improving the image of VET as another training pathway option for students after completing Basic Vocational Training. The educational centres organise inclusive actions both at curricular level and for the students as a whole.
- Changing the social perception of VET by publicising what is done and what is the professional projection from an educational stance. To this end, various activities are proposed for opening the centres to the community, for external dissemination (local, national, and international) of projects, activities, productions by VET students as well as participation in transnational networks of VET teachers.
- Generate experiential learning and socialisation strategies for work. The main aim is to establish synergies with the business sector either through activities with local companies, joint curricular proposals with companies or collaborations with companies to attract talent through 2D games.
- Improve the professional projection and employability of VET students. The guidance departments develop professional guidance actions aimed at how to manage training-labour transitions, increase the expectations of professional employability or provide continuity to projects initiated during the educational period.

Some good practice actions aimed at raising awareness of vocational training are illustrated below (see Table 6).

**Table 6:** AndalucíaSkills 2018.

Professional family: Transport and vehicle maintenance/image and sound/
Development location: IES San José de la Rinconada. San José de la Rinconada (Seville)/ IES Isidoro Sánchez. Málaga/IES La Palma. La Palma del Condado (Huelva)
The competition is aimed at raising awareness and recognising the work of students in Initial Vocational Training, as well as promoting the relationship between companies in the sector and the educational centres. The regional champions of each edition make up the Andalusian delegation that competes in the corresponding Spanskills national championships. The innovative component of good practice is emphasised in the fact that they demonstrate to society that they are prepared and competitive.
**2D game programming**
Professional family: IT and communications
Development location: IES Campanillas. Málaga
Participation of students from Higher Level Training Cycles in integrated projects developing the programming of 2D games through the proposal of challenges, creativity and self-learning. Through the development of 2D games, they have learnt a wide range of advanced programming concepts that help them to acquire greater professional skills and competences. The aim of these workspaces is to collaborate with companies to attract talent through the internal hackathon, thus highlighting the quality of the training provided to vocational training students.
**Building and civil works professional conferences**
Professional family: Building and civil works
Development location: IES Heliópolis. Seville
An experience that arose with the aim of favouring and promoting the relationship between the company and the educational centre, bringing students closer to the reality of working in the building and civil works engineering sector, as well as informing them about the possibility of extending their studies at other educational levels. In addition to establishing synergies with local companies, students from other educational levels of the centre and other related professional families, such as Renewable Energies and Catering, participated actively in this meeting space.

#### Developing active and experiential methodologies in Vocational Education and Training

Actions incorporating active and experiential methodologies in Vocational Education and Training represent 62.5% of the practices analysed in this study. This representative value is evidence of the importance attached to teaching innovation by professionals currently working in this field. These good practices follow a fairly equal distribution in all vocational families with a representativeness ranging from one to three good practices per vocational family, with a total of 8, representing 14.5%. By level, we observe that the incorporation of active and experiential methodologies is more common in Higher level (46.6%), followed by Intermediate level (38.6%) and practically non-existent in Basic vocational training (5.7%).

The analysis of the selected good practices reveals some common lines of work with regard to the incorporation of active and experiential methodologies (see Tables 7 [Table tab8]–9).

- The search for a teaching model that encourages discovery and the development of a critical capacity on the part of students. There is a need to broaden the teaching vision, and to find interdisciplinary models within the content being taught.
- Methodological innovation also seeks to intervene directly in the educational spaces themselves, that is to say, that the knowledge acquired by the students is used to improve them, making them more pleasant environments with their own identity.
- We are moving towards a methodology that promotes the identification of real challenges in the work environment from a social and collective perspective, and along the lines of developing sustainable careers based on ethics and social justice.
- The aim is also to enable students to experience the functions and responsibilities of the different work roles, learning to assume different responsibilities, as well as to demonstrate the implementation of their decisions.

**Table 7:** Flipped classroom in SCA.

Professional family: Transport and maintenance of vehicles
Development location: IES Emilio Canalejo Olmeda (Córdoba, Andalusia)
The experience arises from the need to make the most of classroom time, with the students taking on the leading role as regards their own learning. The aim of the activity focuses on making all learning resources available to students through different media, with these materials initially being worked on independently at home and then in the classroom/workshop. During the class session, the teacher resolves queries about what has been worked on and guides the actions to be performed. The main focus is the students’ active and visible learning. They highlight as an innovative element of the practice the methodology itself as it is considered to be much more productive, fostering self-learning, as well as the active role of the student in the classroom.
**Innovative methodologies. Collective projects: Inter-level pairs/pedagogical pairs**
Professional family: Wood, furniture and cork
Development location: IES La Rosaleda (Málaga, Andalusia)
The project consists of a proposal that acts as the backbone of a collective project of the Department of Wood, Furniture and Cork. This action is approached from the Project Based Learning (hereinafter PBL) methodology to develop the “Wooden Islands” project. All the teaching staff, from their respective modules, participate in the different phases of the process: design, manufacture, finishes and installation. A pedagogical model using “inter-level pairs” from different cycles is used for its implementation. The project involved the participation of 80 students from CFGS, CFGM and FPB, and 28 inter-level pairs. This action aims, on the one hand, to improve the involvement of all members of the Department and to innovate in the classroom through PBL, flexible groups and inter-level pairs.
**Game of FOL**
Professional family: Vocational training and guidance (FOL)
Development location: IES Alfonso XI (Alcalá la Real, Jaén-Andalusia)
This action, inspired by the Game of Thrones books and series, incorporates gamification as the central axis of the teaching-learning process to develop the last training unit of the course, specifically “UT 12: The adventure of finding a job.” The students develop a personal project called “My plan to occupy the Iron Throne,” with this being the only way to be eligible for the job. This action enables learning through play and encourages the commitment of its participants. The educational process has incorporated fictional narratives, videos and vignettes, coins with which to acquire power cards and possible alliances, among other elements.

**Table 8 tab8:** Virtual immersion in art.

Professional family: Image and sound
Development location: IES Profesor Tierno Galván (Seville, Andalusia)
The main objective of this innovative experience is to introduce students to Virtual Reality techniques as a tool for education in general, and specifically for art. The implementation of this experience is developed through interdepartmental cooperation with teachers and students of 1st and 2nd year ESO. Its development aims to foster students’ critical capacity, promote the creation of VR content, increase interdisciplinary-based work at school level and redefine the role of the teacher as a driver of knowledge acquisition in a meaningful manner, and not only as a bearer of knowledge.
**Guadalpín productions**
Professional family: Audiovisual and entertainment production
Development location: IES Galán Guadalpín (Málaga, Andalusia)
This good practice encourages the development of interdisciplinary work and consists of a show in which the entire educational community (teachers, students and AMPA or parents’ association) participates and is held at the Palacio de Congresos in Marbella, open to the entire population and free of charge. Some of the activities carried out include the development of radio programmes in French, reports on the Cultural Week, video and photographic reports on the graduations of the 4th year ESO and 2nd year Baccalaureate students, the school’s graduation photograph, etc. In addition, multi-camera production of situation comedies, tele-plays (adaptations of plays for television), short films and others are carried out.
**#EDUperformance**
Professional family: Vocational training and guidance (FOL)
Development location: IES Tierno Galván (Seville, Andalusia)
This action uses an adaptation of the Design Thinking (hereafter DT) methodology. The process followed consists of five stages: empathy mapping, forced relationships, design, sharing and evaluation. #EDUperformance has four main objectives: to cater for the diversity of talents, facilitate self-learning and self-assessment, create original materials and transform the classroom into a workshop. Among the actions carried out, the following are worth highlighting as innovative procedures: the development of word clouds, questionnaires, image walls and satisfaction surveys on the process followed. In short, they see DT as a highly appropriate methodology for adapting to the diversity of the classroom and adjusting to the abilities and interests of each student

**Table 9:** Start your life: Developing a divergent view in education.

Professional family: Vocational training and guidance (FOL)
Development location: Unidentified IES (Málaga, Andalusia)
This good practice evidences an experiential learning process in which key personal and social competences, such as emotional intelligence and resilience, divergent thinking and creativity and metacognitive reflections, are developed. The development of the teaching-learning process is based on the principle of Bloom’s taxonomy (lower order thinking → higher order thinking) and Edgar Dale’s learning pyramid, which shows the retention rate of information according to the manner in which it is delivered. The aim of this innovation is to encourage cooperative work, develop communication skills, incorporate Service Learning and experiential learning.
**Dual training in bodywork**
Professional family: Transport and maintenance of vehicles
Development location: IES Galán Acosta (Córdoba, Andalusia)
The Dual Vocational Training Project arose with the aim of adapting the training that was being offered to students to the demands made by companies in the area in order to increase the employability of students. The objectives pursued by this experience are: to combine theory and practice throughout the Dual VET cycle in order to “learn by doing,” adapt training to the reality and needs of companies, improve skills and competences, both personal and professional and finally, increase the expectations of professional insertion after obtaining an academic degree. It should be noted that the results obtained show a 100% job placement rate.

#### Strengthening and opening up new avenues for business engagement

Actions considering collaboration between the different guidance counsellors are present in 25% of the selected good practices, which is considered a very low percentage, given the role that companies have in contributing to the development of FCT (Workplace Training) and dual training projects. These good practices are distributed among 14 of the 22 professional families analysed. The families in which no good practice related to the involvement of businesses has been observed are Physical-Sports Activities; Administration and Management; Energy and Water; Personal Image; Installation and Maintenance; Wood, Furniture and Cork; Health and Glass and Ceramics. As far as vocational training levels are concerned, more than half (52%) of the best practices are addressed to both Intermediate level and Higher level. This level is the one that benefits the most, since 24% of the actions are targeted exclusively at this level (thus receiving more than 75% of the good practices). This comes into contrast with 12% of actions aimed at Basic Vocational Training. A few good practices directed at teachers (4%) are also observed.

From the analysis of the good practices selected, a series of axes are derived around which the actions undertaken by the centres are articulated.

- Design and development of didactic units in the centres with the participation of companies in the selection of contents and training activities, the development of these and the criteria and procedures for the assessment of learning, in terms of professional competences. In this way, the curriculum is adapted to companies’ demands for qualified personnel and a space for cooperation, dialogue and joint management is generated between the VET system and the sectoral productive environment.
- Development, in educational centres, of training activities organised with the collaboration of companies (involvement of companies in specific technical training activities for students, collaboration in the development of service-learning projects, development of manufacturing in educational centres with the participation of companies, approaching cutting-edge companies for the development of a didactic unit in the centres, organisation of professional conferences and trade fairs, etc.).
- Carrying out training activities in companies, beyond the Workplace Training module (FCT), aimed at both students and Vocational Training teachers, taking part in technological advances and business organisation.
- Collaboration in the development of plans for analysing emerging market niches, attracting talent, improving job placement opportunities (creation of professional launchers) and promoting business and social entrepreneurship (advice channel for the creation of companies). In this way, companies contribute to improving the visibility and knowledge of VET.

The following (Table 10) are some examples of good practices aimed, among others, at fostering collaboration between educational centres and the business environment.

**Table 10:** Collaboration between educational centre and business.

Professional family: Graphic arts
Development location: IES Llanes (Seville)
Rotating participation of the collaborating companies in the students’ learning by making contributions to the training programmes on different means of production, facilitating the adaptation of the centre’s equipment, presenting innovations in the production process and contributing to the preparation for professional and labour insertion.
**En vivo y en directo (live and direct)**
Professional family: Sociocultural and community services
Development location: C.D.P La Blanca Paloma (Seville)
Aiming to facilitate real experiences with social impact through service-learning. Development of more than 30 events that respond to social demands (associations and institutions in the area) and that are developed in collaboration with companies and third sector entities.
**Business technical seminars/project education**
Professional family: Energy and water; civil works and building; electricity and electronics
Development location: IES Heliópolis (Seville)
Holding technical conferences co-organised by the educational centre, students and companies and aimed at students and the general public. They present the companies’ most significant innovations. Beforehand, the companies work together with the educational centre’s VET students to analyse and understand the innovations.

### Analysis of the relationships between the key elements (objective 3)

The multiple linear regression model associated with career competence development does not have a high coefficient of determination (R = 4.053). However, the overall significance test of the model (*p* = 0.002) indicates that the set of key elements act as predictors of career competence development. A more detailed analysis of the standardised coefficients shows that the “active methodologies” element is the one that explains the average behaviour of the career competences (ß = 0.380; *p* = 0.000). The Chi-square coefficient confirms the significant association between career competences and the use of active and experiential methodologies (Chi-square = 15.757; *p* = 0.000), this being the only association that is significant in relation to this variable.

As far as attention to drop-out rates are concerned, no overall significance was found to indicate that any of the key elements could act as a predictor. However, when studying the model related to the improvement of VET knowledge, an overall significance of the model is reached (*p* = 0.002). Detailed analysis of the standardised coefficients indicates that the elements “attention to drop-outs” (ß = 0.284; *p* = 0.022) and “involvement of companies” (ß = 0.350; *p* = 0.001) explain the average behaviour of VET knowledge. The only variables that reach a significant Chi-square value when associated with knowledge of VET are precisely these two variables [(attention to drop-outs): Chi-square = 5.292; *p* = 0.021; involvement of companies: Chi-square = 10.295; *p* = 0.001].

## Discussion, limitations, and implications

In relation to objective 1, the results indicate that best practices are being developed in VET centres which, despite not incorporating in an integrated manner all the key elements that should be present in comprehensive guidance programmes, may be opening up opportunities to make this possible. It is important to recognise existing resources and opportunities and build on them in order to construct comprehensive guidance programmes, as recommended by Gysbers and Henderson (2014). We have observed that these best practices, however, are coming to life unevenly in the different professional families and levels of VET; hence, it would be necessary to carry out a reflection at institutional level, analyse the causes and promote a more widespread and integrated development.

In relation to objectives 2 and 3, VET must be concerned with training people according to the demands and characteristics of the labour market, not so much of the present, but of the future. The results suggest that VET centres are taking note of this and are taking action to help students develop career competencies that include (ELPGN, 2015) personal management (self-awareness, social and planning skills), learning management (engagement and understanding of the meaning of learning) and career management (information seeking, decision making, coping with transitions). These competences will make students more credible and competent to work and marketable in the increasingly competitive labour market (Ali and Marwan, 2019). The International Centre for Career Development and Public Policy (ICCDPP, 2019) made recommendations to ensure that career-development programmes and services develop citizens’ career management skills all around the world. They recommend that countries identify what career-management skills are important in their context and integrate them into the education system. Some of the literature reviewed in relation to career skills training has argued the need to identify all the possible approaches able to support policymakers, as they can impact key-competence development in different ways and thus benefit or harm more vulnerable populations (Ceschi et al., 2020). Other papers (ETF, 2020) provide examples of best practices developed in the European context.

VET centres try to facilitate the development of these competences through the use of active and experiential methodologies, which also bring students closer to the labour market. This methodological element is one that seems to receive the most attention in VET centres. Linear regression analysis has furthermore indicated that this element is a predictor of career competence development. The ELPGN (2015) acknowledges that a best practice for career development in VET is to provide information and guidance including learning career management skills based on one’s own experience. For its part, VET4EU2 (2018) points out that in order to facilitate experiences that favour a better knowledge of the environment and also of oneself (in interaction with it), broaden their expectations, as well as make more realistic decisions, experiences that bring them closer to the labour market should be offered.

But offering such methodologies alone is not enough. In order to foster students’ career development, it is necessary to establish collaboration networks within and outside the centres. And this is precisely one of the elements that appears most timidly in the best practices analysed. The main difficulty affecting this collaboration lies in the obstacles that arise when coordinating between the different guidance counsellors. In a previous study (Romero-Rodríguez et al., 2020b), it was found a minimal culture of institutional collaboration between career guidance services and school counsellors. Some of the coordinated actions implemented depend on the willingness of the corresponding staff. On the other hand, there is a weak regulatory implementation at regional and national levels that does not allow for deployment of a comprehensive career guidance system. In addition, it was revealed the lack of a clear competence map setting out the roles of the various guidance agents. Barnes et al. (2020) point out, among other obstacles: (a) the different perceptions of the concepts in defining career guidance; (b) the differences in the operating cultures between the different agents and providers of career guidance (education, employment, youth and social administrations); (c) sectoral protectionism that overlaps activities and lack of continuity; (d) the lack of clarity and agreement regarding quality and impact of the services. These obstacles must be overcome and are recommended by the Guidance Resolutions of the Council of the European Union (2004, 2008) which call for ensuring effective cross-sectoral cooperation and coordination of guidance policies. This call is reiterated in various reports published by different international institutions (Vuorinen, 2017), which have also tried to show their collaboration in various joint reports (EU, ETF, CEDEFOP, OECD, ILO, and UNESCO, 2021). Stakeholders participating in a previous study (Romero-Rodríguez et al., 2020b, p. 35) pointed to the following as objectives to be pursued: (1) Design and develop a framework of procedures for coordinating and managing comprehensive guidance actions between administrations, services and guidance counsellors, encouraging participation by the various stakeholder groups; (2) Achieve regulatory implementation of the integrated vocational guidance and information system set out in the current legislation. Some actions proposed in this regard were: (a) working groups (with the participation of the different administrations, services and guidance agents) to draw up a list of collaboration procedures; (b) setting up a telematic system that compiles the guidance actions developed by the different administrations and agents; (c) designing a single virtual platform for the registration of training, professional competences and lifelong guidance procedures for each user; (d) joint work plans and calendars for educational centres, companies and employment agents in the area; (e) continuing professional development plans targeting the various guidance agents, strengthening the focus on integration and collaboration.

One of the concerns of VET centres is the prevention and reduction of ESL. In relation to the various strategies they put in place to achieve this, they have also referred to the exploration of innovative methodologies that foster student participation. Some research studies (Alonso et al., 2018) point out that, by promoting experiential and compensatory methodologies, students at risk of dropping out show a significant improvement in their confidence and academic and personal motivation, which results in them remaining in the system. In this line of improving motivation and meaningfulness in learning, the importance of implementing training activities that link education and work, as well as training in VET skills that are widely accepted in the labour market through the development of participatory action research projects, is highlighted (Tukundane and Zeelen, 2015). However, the results of the analysis carried out indicate that there is still no clear awareness in VET centres of the relationship between this methodological approach and the prevention of drop-outs, nor does it seem to be related to the development of career management competences, as acknowledged in reports published by the Comisión Europea (2015) and the European Lifelong Guidance Policy Network (ELGPN, 2014). There is an association between improving knowledge of VET and preventing drop-outs, which is clearly seen in the actions aimed at raising awareness of VET among secondary education students. Actions to improve the knowledge of VET are carried out within schools and are mainly limited to generating links between the world of education and the labour market and pathways for work-related socialisation (Aiello, 2020; Rintala and Nokelainen, 2020).

Establishing these links requires the involvement of companies. In the Spanish context, a significant evolution of this commitment of companies to VET is being perceived in the named Workplace Training Module (FCT), in the Integrated Schools (CIFP) and in the incipient Dual VET. Companies are increasingly taking a more active role in the design and development of the curriculum and in the co-responsibility for its implementation (Marhuenda-Fluixá, 2019). This role has been claimed by companies in order to promote employability (87% of businesses consider it important in the study conducted by Blázquez et al., 2019). For future research, further analysis of the characteristics of companies involvement is needed (Mann et al., 2018a,b): authentic, frequent and often mandatory, valued, varied, contextualised, personalised and begun at a young age. Others (Gessler, 2017) propose a conceptual framework of collaboration in which a formal structure is defined that must be contextualised by the authors themselves through everyday interactions. Some reports (EU, ETF, CEDEFOP, OECD, ILO, and UNESCO (2021) p. 17) have identified activities that can be undertaken to enhance the collaboration of companies in career guidance: inviting volunteers to schools; career talks; job fairs; mock interviews; CV workshops; workplace visits and observation; internships; businesses competitions; job offers for school leavers; outreach campaigns for professional development; regular career reviews and directing workers to relevant training and internal career opportunities. In these processes, professional tutors play an essential role, for whom specific and contextualised training is required (Domingo Cebrián, 2018; Olmos-Rueda and Mas-Torelló, 2019). For academic tutors, on the other hand, in-company training through internships is called for (Mulder and Winterton, 2017).

Among the limitations of this work, we must consider, firstly, that the internships analysed were presented by the centres themselves. Therefore, there may be other best practices currently being developed in Andalusia’s VET that have not been studied. Secondly, internships have been analysed only on the basis of self-reports prepared by the centres. Future research should use other complementary strategies to identify good practices, as well as using complementary information gathering procedures (interviews with different stakeholders, focus groups, analysis of documents and productions produced by the centres).

We consider that the findings of Villardón-Gallego et al. (2020) in their review of six E2Os (Second Chance Schools) in which several key aspects were identified that could be useful for the development of transversal competencies, would be extensible to the development of career competencies in VET as a whole and open avenue for future research. We look at these experiences, considering that they cater for young people who have dropped out of school prior to completing their education. All the elements we have been analysing in this study are intertwined in these experiences. These include the importance of career competencies for gaining and maintaining employment and the social integration of young people (especially in vulnerable situations); the need to develop transversal competencies through experiential methodologies; ensuring that the learning environment is based on interaction and support (both by businesses and stakeholders). As part of the VET system (e.g., in Spain they are included in Organic Law 3/2022, of 31 March, on planning and integration of Vocational Education and Training), future research should analyse the practices developed in these centres and their contribution to career development and the construction of integrated guidance plans.

## Data availability statement

The raw data supporting the conclusions of this article will be made available by the authors, without undue reservation.

## Ethics statement

The studies involving human participants were reviewed and approved by Ethical Committee for Experimentation of the University of Seville. The patients/participants provided their written informed consent to participate in this study.

## Author contributions

SR-R, CM-M, and TM-B conceived, designed, and wrote together this paper. CM-M wrote the Methods section. The identification of themes was carried out independently by the three researchers. SR-R, CM-M, and TM-B analysed and interpreted the data. All authors described the theoretical framework and wrote the results and discussion sections. All authors contributed to the article and approved the submitted version.

## Funding

The first phase of this project (Objective 1 - best practices identification) has been funded by Dualiza Caixa and supported by Junta de Andalucía (Spain) Project Career Guidance in Andalusia: Situation and proposals for Vocational Education and Training (PRJ201903650; CGT: 0399). Objectives 2 and 3, literature review, data analysis and interpretation and discussion have been developed as a part of the project “Early leaving of Vocational Education and Training: Design of a comprehensive career guidance system (Orienta-2O)”, co-financed by funds from the Andalusia ERDF operational programme 2014-2020 and by Consejería de Transformación Económica, Industria, Conocimiento y Universidades de la Junta de Andalucía (Regional Ministry of Economic Transformation, Industry, Knowledge and Universities of the Andalusian Regional Government) (Project reference: P20_01131).


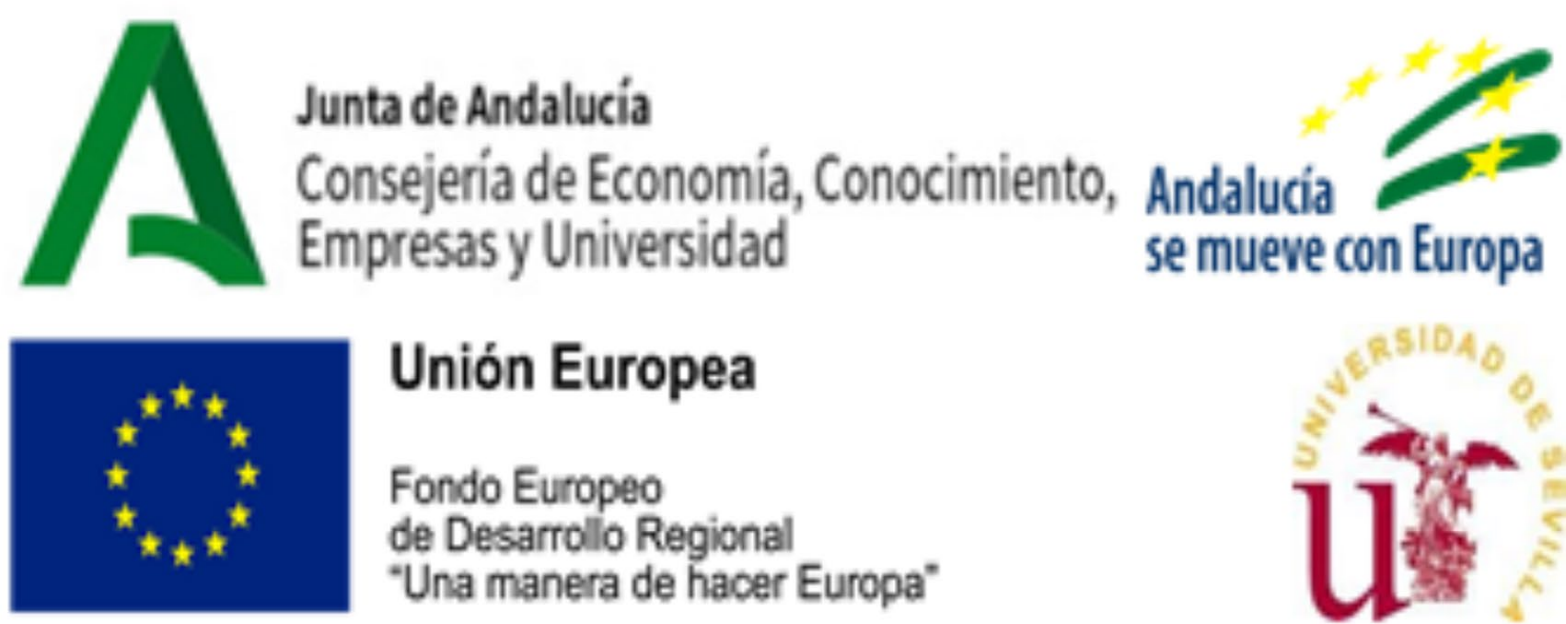


## Conflict of interest

The authors declare that the research was conducted in the absence of any commercial or financial relationships that could be construed as a potential conflict of interest.

## Publisher’s note

All claims expressed in this article are solely those of the authors and do not necessarily represent those of their affiliated organizations, or those of the publisher, the editors and the reviewers. Any product that may be evaluated in this article, or claim that may be made by its manufacturer, is not guaranteed or endorsed by the publisher.

## Footnotes

^1^ https://www.cedefop.europa.eu/en/tools/vet-toolkit-tackling-early-leaving

^2^ https://worldskills.org/
